# Pediatric medication management barriers faced by providers serving underserved populations and proposed solutions

**DOI:** 10.3389/frhs.2025.1569531

**Published:** 2025-06-12

**Authors:** Tiranun Rungvivatjarus, Maria Z. Huang, Britanny Winckler, Erin S. Fisher, Kyung E. Rhee

**Affiliations:** ^1^ Department of Pediatrics, University of California San Diego, San Diego, CA, United States; ^2^ Division of Pediatric Hospital Medicine, Rady Children’s Hospital, San Diego, CA, United States; ^3^ Department of Pediatrics, University of California Irvine, Irvine, CA, United States; ^4^ Division of Hospital Medicine, Children’s Hospital of Orange County, Orange, CA, United States

**Keywords:** medication, health literacy, pediatric medication management, medically underserved area (MUA), health professional shortage areas (HpSA)

## Abstract

**Background:**

While families with limited English proficiency and/or low health literacy face increased risks of medication errors, poor medication management, and non-adherence, little is known about the barriers pediatric medical providers experience when educating families on medication use and compliance. This study explores provider-reported barriers and proposed solutions to improving medication management.

**Methods:**

Focus group discussions were selected to obtain data as they can provide rich insight into participants’ perspectives. From December 2019 to September 2020, focus group discussions were conducted with pediatric providers from four clinics located in Medically Underserved Areas/Medically Underserved Populations/or Health Professional Shortage Areas, which are geographic areas/populations with limited primary care access. Initially held in person, focus groups shifted to virtual formats during the COVID-19 pandemic. Two investigators independently coded each focus group in an iterative process and worked with a third investigator to discuss and refine emergent themes via inductive thematic analysis.

**Results:**

Sixteen providers participated across four focus groups. Four major themes were reported as barriers: (1) time constraints, (2) provider's lack of medication and logistical knowledge, (3) incomplete or absent patient medication information, and (4) complexity/inefficiency of the electronic health records system (EHR). Providers identified three possible solutions to such barriers: (1) EHR optimization or technology tools, (2) dedicated medication educators, and (3) video/graphic tools.

**Conclusion:**

Pediatric providers face barriers in supporting families with medication management including lack of time, knowledge, information, effective EHR. Proposed solutions such as improved technology, dedicated educators, and visual aids may help address these issues while potentially decreasing medication errors.

## 1 Background

Patients and families with limited English proficiency (LEP) and/or low health literacy (LHL, defined as the degree to which individuals have the ability to gather, comprehend, and use information to make health decisions (1) are at greater risk of experiencing medication errors and poor medication management, which can lead to worse health outcomes (2–8). For example, parents with LHL had lower mean scores on asthma knowledge questions when compared to those with higher literacy (9). More importantly, children of parents with LHL were more likely to have emergency department (ED) visits, hospitalizations, and missed school days due to asthma compared to children in the higher parental literacy group (9). Thus, improving pediatric medication management and adherence is an important area of study for pediatric health.

Accurate medication management is challenging for several reasons. Rothman et al. found that healthcare providers often do not provide clear medication dosing, frequency, and treatment length details. Furthermore, the US Food and Drug Administration medication guides are typically written at tenth-grade reading levels, which is more advanced than the American Academy of Pediatrics’ (AAP) recommendation to use patient educational materials at a sixth-grade level (4, 10). Additionally, clinicians or pharmacists rarely provide standardized medication dosing instruments despite the AAP's endorsement. Thus, many caregivers continue to use non-standardized utensils for administration of liquid medications (4, 11).

While these challenges highlight patient barriers to medication management, there are few pediatric studies identifying healthcare provider barriers (12–15). Pediatric healthcare providers (PHP) have a central role in counseling, prescribing, and facilitating medication adherence for pediatric patients, yet they may face significant barriers to helping families with LEP/LHL manage their child's medications. The goal of this study was to better understand the challenges medical providers face in helping families manage their child's medications and the potential solutions PHPs propose. With an enhanced understanding of barriers and solutions, future studies will be able to tailor community interventions, education, and resources to best serve this at-risk population.

## 2 Methods

### 2.1 Study setting

Focus group discussions were conducted between December 2019 and September 2020 in San Diego, CA. We recruited PHPs from 4 community clinics located in Medically Underserved Areas (MUAs), Medically Underserved Populations (MUPs), or Health Professional Shortage Areas (HPSAs), as defined by the Health Resources & Services Administration (16). MUAs are geographic areas with limited access to primary care services, MUPs are groups of individuals who lack adequate access to primary care services, and HPSAs are designated regions experiencing a shortage of healthcare services. These designations identify geographic areas and populations facing significant barriers to healthcare (17). We chose to recruit healthcare providers from these areas because area-level disadvantage and health literacy are closely correlated, suggesting that many patients in these clinics have LHL or LEP (18).

### 2.2 Participants and recruitment

Four community clinics in MUA/MUP areas were identified and targeted for recruitment. Participants were eligible if they provided pediatric care in underserved areas or clinics designated MUA/MUP and were likely to care for families with LHL/LEP. Two of the community clinics served both adults and children while two clinics served only children. Two of these clinics were also teaching clinics with family and pediatric medicine residents. None of the clinics had an on-site pharmacy, and all of the clinics used an electronic health record (EHR)—Epic Systems Corporation, CMIS Medical EHR V2.0—to prescribe medications. PHPs (physicians, registered nurses, and nurse practitioners) involved in pediatric medication management at these four clinics were eligible to participate in the study. Our research team had an existing partnership with the lead physician at each clinic, who helped distribute study opportunities to their colleagues via e-mail. The University of California San Diego Institutional Review Board approved this study.

### 2.3 Data collection

We conducted focus groups both in-person at the clinics and, during the COVID-19 pandemic, via virtual video conference (Zoom Video Communications Inc., San Jose, California). Each participant signed a written informed consent and received a gift card of $25 for their time and effort. Two research members facilitated PHP discussions (TR and MH, both trained in qualitative interviewing techniques), and all focus groups were conducted in English. The focus groups ranged between 2 and 4 participants. Prior to each session, participants completed a demographic questionnaire, which assessed providers’ clinical role, years of employment, frequency with which they encounter families with medication issues, frequency with which they educate families about medications, and types of barriers to medication education. The order, question structure, and format of the discussion guide were informed by research team members with qualitative research experience. Focus group questions queried PHPs’ barriers affecting medication management and proposed solutions (Supplementary Material). We allowed up to 90 min of discussion during each focus group. The primary facilitator led the focus group discussion while a second facilitator observed body language, noted group responses, and asked probing questions. Focus group audio recordings were transcribed verbatim by research team members (TR and SC).

### 2.4 Data analysis

Data were collected through demographic questionnaires and focus group discussions. Questionnaire responses were presented as percentages and counts where applicable. Inductive thematic analysis was utilized throughout the review of the focus group transcripts (19). Two investigators (TR and MH) independently reviewed the first PHP focus group transcript, coded emergent concepts, and created independent codebooks. Then, they met to discuss findings and to consolidate and refine the codebooks into a single unifying codebook. After independently reviewing the second PHP focus group, the investigators met again in addition to a third investigator (BW) to review discrepancies and reach consensus in coding and to discuss emergent themes. The codebook was updated in an iterative process throughout transcript review. After all transcripts were independently reviewed and coded using the updated codebook, the research group met again to discuss emergent concepts and overarching themes. Thematic saturation for PHP factors affecting medication management was reached after four focus groups. Themes reported in PHP focus groups that were specific to parental barriers (e.g., limited health literacy, forgetfulness) were analyzed and reported separately (12). Demographic questionnaire responses were presented as percentages and counts where applicable. Qualitative data analysis software, Atlas.ti Scientific Software Development GmbH©™ (Germany), was used to organize and analyze transcripts.

## 3 Results

Sixteen PHPs participated across four focus groups. Table 1 summarizes provider characteristics and questionnaire responses. Most participants (87.5%) were physicians. Seventy-five percent of participants reported encountering families with medication issues at least a few times a week. The majority (68.7%) of participants performed medication education greater than once daily. Thematic analysis revealed four PHP barriers to facilitating medication management for families living in underserved communities (Table 2); three proposed solutions were identified (Table 3). Participant job titles were withheld for each of the supporting quotes to maintain anonymity given the small sample size.

**Table 1:** Characteristics of pediatric healthcare providers (*n* = 16).

Characteristics	(*n*, %)
Professional role	
Pediatrician/family medicine physician	14 (87.5)
Registered nurse/LVN	1 (6.2)
Nurse practitioner	1 (6.2)
Years of employment	
<1 year	2 (12.5)
1–5 years	5 (31.3)
6–10 years	4 (25)
11–20 years	2 (12.5)
>20 years	3 (18.8)
How often do you encounter families that have issues with their child's medication(s)?	
Never	0 (0)
<once a week	3 (18.8)
About once a week	1 (6.2)
A few times a week	8 (50)
Once daily	1 (6.2)
>Once daily	3 (18.8)
How often do you educate family members about their child's medications?	
Never	0 (0)
<once a week	0 (0)
About once a week	0 (0)
A few times a week	5 (31.3)
Once daily	0 (0)
>Once daily	11 (68.7)

**Table 2:** Themes and supporting quotes on provider barriers to medication management.

Themes	Quotes
Time constraints	“I think I am medicating appropriately…but I don't stop and say ‘do you [parents] understand what I told you?’ because I don't have time to repeat myself a bazillion times, so I think it is time [that's the issue].” “A lot of time I didn't have that opportunity because it is a very busy day and didn't have time to explain. You bring [parents] back for a follow-up especially if it [involves] chronic medication like for asthma.” “It can be really hard… our clinic is supposed to be super fast. You're only supposed to spend 15 min with them so when parents are coming in and asking these [medication-related] questions, it is hard to fit it all in when you have other people waiting.”
Provider's lack of medication and logistical knowledge - Pharmacy-related information - Alternative or Herbal Medications - Specialty or New Medications	“I don't know the shelf life of certain medications so I ordered a month's [worth of medication] and pharmacy gave [families] a month [supply], but then [pharmacy] said [the medication] only lasts for 15 days. So then they're throwing [medication] away, and I'm reordering because there's not enough refills.” “The other part of the medications piece is the ‘grey' medications – the symptomatic relief over-the-counter, herbal supplement, the Zarbee's, the non-prescription medications that parents can sometimes be giving to their kids daily or very often. I certainly often don't have too much of a clue of what exactly they do. That's when I resort to literally Googling the name and getting the pictures, and try[ing] to look at the ingredients label because I have no idea.” “…people who ask us to refill their psych meds because they can't get in to a provider. At some level, I'm a little uncomfortable. When it is a [new] patient who is on multiple medications, I start getting uncomfortable about the counseling because I don't normally prescribe that many psychiatric medications to one person, or I don't use [some] of these psychiatric medications so I don't know as much about the side effects.”
Incomplete or absent medication information	“Parents coming in with already trying to treat their kids with expired medications or getting medications across the border. For us [providers], trying to figure exactly what those medications were, what the indications were,…[and then] there's also an added language barrier. Things like that can be difficult sometimes.”
Complexity/inefficiency of the electronic health records system	“I think there is a way to be able to see within Epic® (EHR system) if they have been picking up medicines, but I don't think there is a way [to know] they didn't pick it up, a warning kind of a thing. So, the EMR probably could be a little better.” “I would say that our current system of having parents communicate with the office for medications is somewhat lacking in that there really is no easy way for them to send a picture of the medication. Parents have to go through MyChart (electronic patient portal) and then they have to know where to log in, … figure out how to get a picture on their camera into their computer to then send.”

**Table 3:** Themes and supporting quotes on providers' proposed solutions.

Themes	Quotes
Electronic health records optimization or technology tools	“Since we sign up all of our families into MyChart, maybe [medication administration instructions] could be built into MyChart. We could build it into one of those apps in the system so that it is an easy way that everything is connected.” “Better ways for families to navigate medicines and communicate [medication information to others] would be really helpful. So, some type of portable medical record, or an educational piece that they could have that would be kept up [to date] so that it could be communicated to people that need to know.” “If there was a way to tag in that chart ‘this patient needs a follow-up call about their medications,’ because not everybody would need one, to see were [the patient] able to get the medicine?”
Dedicated medication educator	“I know other organizations have health aides or health educators for the chronic illnesses. When the patients come in [to clinic], they watch a video and they spend that time reviewing all of the medications.” “In asthma for example, I think you [can] have someone at the level of a medical assistant that happens to be specially trained, who knows the information and could convey [medication information] to families.”
Video/graphic tools	“I guess ideally if we could have pictures of their medication and name underneath it so that [families] have a spreadsheet of what their child is taking, the dosage, or the most common side effects all on one page.”

### 3.1 Provider barriers

#### 3.1.1 Time constraints

Providers noted insufficient time in clinic to provide education or answer medication-related questions. Many mentioned scheduling families for a follow-up appointment if unable to address medication issues in the time allotted. When there were multiple issues to discuss during the follow-up appointment, providers also expressed the need to prioritize discussion topics as medication-related topics often take a long time.

“You are in a room for a period of time trying to triage what you want to spend your time on, and [medications] are the kinds of things that can just suck you in, really [it's] time that could be probably use[d] in better ways.”

“It requires a longer [medication] educational discussion which sometimes can be difficult in a busy pediatric practice to be able to have that time to sit with families and make sure that they truly understand fully.”

Another barrier related to time was providers’ lack of time to review medication information or educate themselves about unfamiliar medications.

“When a new patient comes, we inherit a medication list. I am being asked to refill medications that I'm not as familiar with. So again, creating time…to educat[e] myself or ask [parents] how comfortable they are giving it if they know enough about the medication.”

Time constraints can hinder PHPs’ ability to effectively help families manage their child's medications, especially when reiteration or extensive education is needed to ensure parent comprehension.

#### 3.1.2 Provider's lack of medication and logistical knowledge

Providers identified their lack of knowledge about a medication as a barrier to helping families manage their child's medications. PHPs sometimes perceived that for some medications (particularly those prescribed by a sub-specialist or new medications), they had insufficient understanding of or limited access to information on medication indication, delivery, and side effects. This deficiency led to discomfort in counseling or assisting families. Specifically, as general pediatric practitioners, they reported a lack of comfort discussing, prescribing, or refilling subspecialty medications.

“If it’s a medication that I don’t often prescribe and now I am being asked to refill medications that I’m not as familiar with, technically I should be educating about risk and benefit [but] I don’t always know.”

“You know as new medications come out, [I] try to figure out how I should educate myself, you know, to just stay up with new medications and what we should be saying about those particular meds.”

Providers reported also feeling deficient in knowledge around alternative/herbal medications or products derived from plants, herbs, or other natural sources that are often considered alternatives to conventional pharmaceutical drugs.

“Alternative medicine can also be hard. For some families…like Indian families, Ayurvedic medicine that they will take or their grandparents tell them to take will have interactions with other things. So knowing what they are is the first step…I find myself googling…‘what is this medicine?’”

A commonly reported area of low proficiency was general pharmacy-related knowledge, which included topics such as pharmacy logistics, insurance, and common pharmacist counseling information (e.g., medication storage and timing around meals).

“Agree with [provider] A, [I don’t know] where [medications] should be kept. I got a call one weekend about a mom leaving amoxicillin out on the counter. I was like, 'should be fine?’ I told the nurse triage to call mom back to just call the pharmacy.”

“Sometimes we don’t know which pharmacies are 24 h, and sometimes the families don’t even know. And when I do evening clinic, it's like, well I don’t know! Do you pick it up tonight? Was it sent over? and sometimes they’re trying to change it and it's like, that's a time sink too.”

Pediatric clinics located in underserved communities face a multitude of challenges as demonstrated above, including logistical issues such as pharmacy access and insurance. Additionally, the rapid progress in medicine poses a challenge as providers must continually update their knowledge around new medications to provide relevant information to parents/caregivers.

#### 3.1.3 Incomplete or absent medication information

Incomplete or absent medication information from missing or inaccessible patient health records made it difficult for providers to assist families with their medication questions. Partly due to families’ inability to recite or access their child's medication list, providers found themselves guessing what medications the child was taking.

“It’s sometimes a guessing game of ‘what could this provider have been thinking?’ to have prescribed this combination of medications that I think that [parents are] describing, but I’m not certain that [the child is] on.”

“When you add a [medication] order, you have like 20 million options…[If you] don’t have the [written label] in there, because sometimes [family] can just add in a medication but you don’t put in the dosing or the frequency, the family doesn’t know this, so then you have to add it yourself, and sometimes you just don’t know.”

As demonstrated above, missing information was not limited only to medication names, but also to dosing, frequency, indication, and clinical reasoning. This highlights the importance of accurate and accessible medication lists in patient medical records.

#### 3.1.4 Complexity/inefficiency of the EHR

Providers also reported struggling to navigate quickly or easily through the intricate and multifaceted EHR software system to perform necessary patient care tasks.

“If [patients] get seen at an outside urgent care or emergency department, sometimes Care Everywhere can’t quite link the two [EHR systems] together, even though they should be able to. It's just hard to guess what medicine patients might be on if they saw an adult provider.”

Limitations of the medication prescription system in the EHRs often prompted workarounds that could be more time-consuming, error-prone, or even equally ineffective.

“Sometimes the [prescription] sig doesn’t let you type as much as you would like to, and so then you’re trying to put it in the After-Visit-Summary (AVS), but I’m worried they’re going to lose the AVS. Sometimes I wish there was more room for me to type it somewhere or I don’t know if the notes to pharmacy will show up.”

Overall, these limitations underscore the need for enhancement of EHR systems and patient communication tools to improve medication lists and instructions.

### 3.2 Proposed solutions

#### 3.2.1 EHR optimization or technology tools

Providers expressed how EHR system optimization and new technology tools could assist them in their efforts to help families manage their child's medications. In particular, providers envisioned technology seamlessly integrating into daily patient routines to enhance their medication adherence and safety. Examples included “smart refill bottles” that alert families when refills will be needed or medication applications that send reminders to caretakers when a child's medication is due.

“I mean I don’t know if there’s something out there – some smart refill bottles that could tell you when your medication’s running low, or you know something along those lines… or at least a reminder. A reminder that says ‘Oh, time to take your medication.’”

One provider also proposed an EHR warning that alerted them if a patient had not picked up their medication.

“I think that is a key component to improving compliance in having staff that can educate and encourage parents to do the right thing. It could be at any level to check on whether they are compliant or not. It could be as simple as someone [calling to say] ‘Hey we noticed you got out of the hospital last week. Are you still taking [medication] A, B, and C?’ If you have somebody doing that on a routine and reminding them. It could be helpful.”

Another opportunity for EHR technology optimization was its ability to serve as a common platform for information to providers and patients.

“The more you can integrate the prescribing system so that just the scripts are visible to providers, would be very useful. Just being able to pull in prescriptions or see prescriptions that were prescribed in urgent cares or emergency rooms, specifically, would be already incredibly much more helpful.”

Providers suggested having the ability to incorporate medication administration instructions, interactions, education, and side effects automatically into the patient's AVS or online patient portal, allowing multiple individuals involved in the child's care to have the most accurate information. This proposal also included a platform in which medications prescribed in various healthcare systems could all be aggregated in one place for providers and families. They emphasized the importance of connectivity within the healthcare system to streamline communication and facilitate information sharing between providers and patients.

#### 3.2.2 Dedicated medication educators

There was a strong emphasis on the importance of patient education and empowerment in medication management. Providers advocated for structured educational programs, classes, or resources where families could learn about their medications including their purpose, administration, and potential side effects. Providers suggested leveraging healthcare personnel, such as educators, health aides, or medical assistants with specialized training, to convey medication information to families. These individuals could play a crucial role in empowering families to make informed decisions about their child's healthcare. While some providers proposed education outside of the clinic settings, especially for long-term medications, others expressed value in providing personalized instruction or resources to families during their clinic appointments. This approach could help medication education be accessible and tailored to the patient's specific needs and circumstances.

“You would say [to families] ‘we have identified you as a new asthmatic. You know we have a Tuesday 5–7pm [class] in this office, and you can learn more about asthma, and [the asthma educator] could educate you.’ [Families] will learn about all the preventatives. Which is the quick acting [medication]? Why you would use this [medication]?”

In certain cases, providers highlighted the importance of a multidisciplinary approach to patient care, where various healthcare professionals (case manager, social worker, pharmacist, physician, and nurse) would collaborate to address medication management and education. This approach would create comprehensive support for patients with chronic illnesses, such as asthma or eczema, and facilitate better understanding and adherence to medication regimens.

#### 3.2.3 Video/graphic tools

Providers emphasized the potential benefits of utilizing technology, such as animated applications or videos, to educate patients about medication management. Visual aids could enhance children's and parents’ understanding through clear and engaging demonstrations of medication administration techniques and dosing schedules.

“Maybe having some animation related apps or things that could help [families] even when they go home if they forgot…an animation [showing] this is how you take your antibiotic or this is how many days [you take it for]. If [videos] had something common that both parents and kids [can enjoy], [I] would like that.”

Animations, drawings, or spreadsheets could convey medication instructions at an appropriate literacy level, which could help caregivers remember dosing schedules and medication administration techniques.

## 4 Discussion

In this study, we successfully identified key barriers that may diminish a PHP's ability to provide optimal medication management to families and uncovered solutions to such barriers. To our knowledge, this is the first qualitative study identifying pediatric provider barriers to medication management in underserved communities that typically have higher numbers of families with LEP and/or LHL. Given that medication issues are a significant, multifaceted problem and PHPs are responsible for much of the medication prescribing and counseling, our findings may help shape future resources and interventions to support pediatric providers while increasing medication safety in at-risk populations.

Time constraints have been a well-documented barrier for providers, noting that time for patient education on medications and adherence strategies are rushed or curtailed on busy clinic days (20–24). Similar to the pediatric providers in our study, general adult practitioners felt they had insufficient time to review all the necessary medication details (25). While our study is the first to demonstrate time constraints as a barrier to medication education in pediatrics, time has been a limitation in other areas of pediatric education such as vaccination promotion and confidential teen interviews (26, 27). When providers educate patients about medications and adherence strategies, patient cognitive fatigue, exacerbated by long appointment waiting times, may limit patient comprehension and application to successful medication delivery (21). We can extrapolate a similar scenario to parents receiving education on their child's medications, particularly if they are already managing their own stress and responsibilities.

In our study, PHPs proposed dedicated medication educators and other resources to potentially alleviate time constraints. Assigning medication education tasks to dedicated personnel such as pharmacy technicians embedded in the office could help distribute provider workload, ensuring patients receive comprehensive medication guidance (28). Other solutions to consider include group education sessions to address chronic disease medications or extended clinic appointment times, all of which could potentially alleviate providers' time pressure.

Various technology solutions were suggested to improve medication management. For example, mobile applications have been helpful in providing refill reminder as well as medication information and adherence alerts (29). Automated text message/phone call reminders have been shown to increase medication adherence rate (30, 31). This could be particularly effective in teenagers and young adults suffering from chronic diseases that require long-term medications, such as asthma or diabetes. These technological tools could provide education on medication delivery techniques, storage questions, and several other topics prompted by a caregiver. By creating a resource outside of the clinic setting, caregivers can access this information in real time when they need it, and healthcare providers can limit the extent of these conversations in the office setting.

Lastly, medication event monitoring systems can track the opening and closing of a medication bottle or inhaler use, allowing providers to objectively measure adherence (32, 33). Such tools have demonstrated effectiveness in pediatric migraine, cystic fibrosis, and epilepsy (32, 34, 35). With patient use of medication event monitoring systems, providers would be able to access objective compliance data, which, in turn, can inform the development of patient-specific interventions to improve adherence. Despite lack of consensus on the superiority of a single technology to deliver medication education, increase adherence, and optimize providers' time, it is crucial that providers are educated on the available tools and resources. Undoubtedly, future studies performing a full economic evaluation including a technological tool's time saved or return on investment are much needed.

In today's healthcare environment, technological tools and EHR systems present a paradox in medication management for physicians. While various challenges can make them one of the biggest barriers, they also offer some of the most effective and powerful solutions when properly utilized. In our study, medication adherence was a barrier reported by our PHPs despite the fact that there are over 70 applications of medication adherence monitoring technology available. The lack of interoperability of these monitoring technology systems with established clinical information systems and workflow creates a new barrier to medication adherence (36). Our finding highlights the need for software developers to consider the implications of medication adherence data captured in clinical settings and interoperability of technological tools for pediatric providers.

Another challenge highlighted in our study was the gap in medication and logistical knowledge among PHPs. Both adult and pediatric studies have reported this barrier with disease-specific medications (e.g., hypertension, prophylactic HIV treatment, sickle cell disease, asthma, and obesity) (23, 37–42). Our study highlights that now is an opportune time for general pediatric medication topics (including storage, administration, and common alternative/herbal medications) to be incorporated into future continuing medical education (CME) programs. This is particularly important for providers working in under-resourced areas with LHL populations because these providers often need to balance evidence-based practice with feasibility for the patient. Readily available education can empower providers to better support their patients and families. Furthermore, training and resources on cultural competency, health literacy, and social determinants of health for pediatric trainees and providers can help them develop a more nuanced understanding of the complex factors that influence healthcare access and utilization.

While we reported on providers' perspectives of barriers and facilitators to improving medication knowledge and adherence, our study is not without limitation. We did not specifically query whether each provider sees patients with LHL or LEP. We chose to recruit participants from clinics located in the MUAs/HPSAs because studies have shown that area-level disadvantage and health literacy are closely correlated (18). The generalizability of our findings may also be limited to underserved populations; however, we believe our study contributes to an increased understanding of the complexity of pediatric medication management in LEP and LHL communities. Lastly, given that part of our study occurred during the COVID-19 pandemic, providers' perceptions of barriers, clinic flow disruption due to staffing shortages, and rapid shift toward telehealth services may have affected the topics discussed during the focus groups. These themes, however, remain applicable during the post-pandemic era and should help prioritize target areas and community resource allocation to support PHPs.

## 5 Conclusion

Pediatric providers serving LEP and LHL communities identified several barriers to helping families manage their child's medications, including time constraints, provider's lack of medication and logistical knowledge, incomplete or absent patient medication information, and complexity/inefficiency of the EHR. Three proposed solutions (EHR optimization or technology tools, dedicated medication educators, and video/graphic tools) offer promising future interventions to help providers overcome these challenges and enhance their capacity to assist and educate families effectively. By addressing these barriers, we can improve pediatric medical providers' medication management skills, thereby supporting the health and well-being of pediatric patients and reducing the potential for medication errors.

## Data Availability

The raw data supporting the conclusions of this article will be made available by the authors, without undue reservation.
