# Supplementary material for: Pediatric medication management barriers faced by providers serving underserved populations and proposed solutions
Source: Front Health Serv. 2025 Jun 12;5:1569531. doi: 10.3389/frhs.2025.1569531 (PMC12198145; doi:10.3389/frhs.2025.1569531)
Supplement: Supplementary file 1 [file Table1.docx]

Supplemental Material

Intro: Thank you for taking the time to participate in this discussion. You are all here because you are a on a medical staff who cares for children who take medications. We are interested in your experience with caregivers and family members who manage these mediations. Our focus will be on education and resources associated with medications. While insurance issue is a problem that all providers face, that topic will not be our focus in our discussion today. All of your thoughts are welcome. There are no right or wrong answers; we are simply interested in your thoughts. Since we are going to be recording, please speak up and respect each person’s turn as they speak. Please state your #/letter prior to speaking and if you address other participants, use their #/letter. If you have any questions or if something is not clear, please feel free to ask for clarification.

1. What barriers do YOU face in being able to provide families with medication education/resources?
   1. Time? Language barrier? Multiple family members? Family’s receptiveness? Knowledge? Lack of protocol or standard?
2. How do you think these medication problem(s)/concern(s) affect your patients? Patient’s health? Patient’s family?
   1. Does it affect your patient’s recovery from the illness? how long it takes to recover? Go back to school? Return to his/her normal activity?
   2. Does it affect your patient’s risk of having to go back to the hospital or the emergency room?
   3. Does it affect family time? family’s daily routine?
   4. Does it affect family member work? Time off work? Or work schedule changes?
3. If you were to create a new community resource to help families manage their child’s medication, what would it look like?
   1. Types of information
   2. In what form: electronic/live person/paper? Where it would be stored
   3. Additional functionality?
4. What are the roles of pediatric medical staff when it comes to patient’s medications?
   1. Should the medical staff provide education and resources? Provide troubleshooting support? Provide insurance support?
   2. If so, should a particular type of staff provide such support? (MD, MA, RN, NP, PharmD)
5. Is there anything that we should have talked about but didn’t?
